# Cost-effectiveness of increased contraceptive coverage using family planning benefits cards compared with the standard of care for young women in Uganda

**DOI:** 10.1186/s40834-022-00206-8

**Published:** 2023-02-13

**Authors:** Elly Nuwamanya, Joseph B. Babigumira, Mikael Svensson

**Affiliations:** 1 Infectious Diseases Institute, College of Health Sciences, Makerere University, P. O Box 22418, Kampala, 40530 Uganda; 2 GHE Consulting, P.O Box 27011, Kampala, Uganda; 3 Department of Community Medicine and Public Health, Sahlgrenska Academy, University of Gothenburg, P. O Box 414, 40530 Gothenburg, Sweden; 4 Impact Health LLC, 8287, O’Brian Ave NE, Otsego, MN USA

**Keywords:** Contraception, Cost-effectiveness, Decision-analytic model, FPBC, Decision tree

## Abstract

**Background:**

Uganda has a high population growth rate of 3%, partly due to limited access to and low usage of contraception. This study assessed the cost-effectiveness of the family planning benefits cards (FPBC) program compared to standard of care (SOC). The FPBC program was initiated to increase access to modern contraception among young women in slums in Kampala, Uganda.

**Methods:**

We developed a decision-analytic model (decision tree) and parameterized it using primary intervention data together with previously published data. In the base case, a sexually active woman from an urban slum, aged 18 to 30 years, was modelled over a one-year time horizon from both the modified societal and provider perspectives. The main model outcomes included the probability of unintended conception, costs, and incremental cost-effectiveness ratio (ICER) in terms of cost per unwanted pregnancy averted. Both deterministic and probabilistic sensitivity analyses were conducted to assess the robustness of the modelling results. All costs were reported in 2022 US dollars, and analyses were conducted in Microsoft Excel.

**Results:**

In the base case analysis, the FPBC was superior to the SOC in outcomes. The probability of conception was lower in the FPBC than in the SOC (0.20 vs. 0.44). The average societal and provider costs were higher in the FPBC than in the SOC, i.e., $195 vs. $164 and $193 vs. $163, respectively. The ICER comparing the FPBC to the SOC was $125 per percentage reduction in the probability of unwanted conception from the societal perspective and $121 from the provider perspective. The results were robust to sensitivity analyses.

**Conclusion:**

Given Uganda’s GDP per capita of $1046 in 2022, the FPBC is highly cost-effective compared to the SOC in reducing unintended pregnancies among young women in low-income settings. It can even get cheaper in the long run due to the low marginal costs of deploying additional FPBCs.

**Trial registration:**

MUREC1/7 No. 10/05-17. Registered on July 19, 2017.

## Background

Uganda’s population of 45 million grows at an annual rate of 3.34%, one of the highest growth rates in the World [1]. This is, at least in part, due to limited access to and use of modern contraceptive methods [2, 3]. Like in many low-income countries, women in Uganda face many challenges and risks related to sexual and reproductive health, such as a high unmet need for contraception (approximately 30%), which leads to 43% of all pregnancies being unintended. Low contraception use also leads to a high rate of abortion and unsafe abortion: 30% of all abortions in Uganda are unsafe [3]. Other barriers to using contraception include fear of side effects, infrequent sex, financial incapability, ignorance about methods, and partner opposition towards contraception [2, 4]. Among women with an unmet need for contraception, the largest proportion is poor and less educated [2], predominant characteristics of an urban slum population.

Despite the slowly diminishing unmet need for contraception, more work is needed to ensure that all women, irrespective of their background and socio-economic status, have access to modern contraception methods and are sensitized, along with their partners, about the costs of unintended pregnancies and unplanned families [2]. This aligns with the view that everyone has the right to health under article 25 of the Universal Declaration of Human Rights [5]. Fulfilling the unmet need for contraception in Uganda would have substantial benefits, including averting an estimated 76,000 annual maternal deaths, saving $8.3 billion in costs of unintended pregnancies, and generating net savings of about $7 billion that would have been spent on child and maternal care [6]. This is also fundamental to the achievement of sustainable development goals (SDGs) 2 and 3 (zero hunger and good health and wellbeing) [6]. Despite the evidence of high returns of investing in contraception, Uganda has not prioritized access to contraception [7].

The inequality of access to modern contraception is a reality not only in low-income countries but also in high-income countries. Different studies have shown that the utilization of long-term modern contraception methods varies across the area (region of residence), age, and socioeconomic status – with poor access among low-income earners, the unemployed, and rural dwellers [8–14]. The question researchers and policymakers worldwide should be asking is, what interventions or strategies are currently in place to reduce inequalities in accessing modern contraception?

Several innovative service delivery approaches have been pilot-tested to increase contraception uptake, including community outreaches, mobile phone applications, youth corner spaces, social marketing, and franchising and community-based distribution [15–18]. Voucher-based initiatives are a feasible means of increasing contraception usage in low-and middle-income countries (LMICs) [19–22].

Economic evaluation is the comparative analysis of two or more healthcare interventions in terms of both their costs and benefits (outcomes) [23]. Many contraception interventions in LMICs have been assessed and reported to be cost-effective [24–28]. Similarly, many cost-effective contraception interventions have been documented in Uganda [29–33]. However, no study has been conducted regarding incentive-based contraception interventions in Uganda.

In this study, we assessed the one-year cost-effectiveness of using an incentive-based family planning benefits card (FPBC) to increase access to contraception services among young women in slums in Kampala, Uganda, compared to the standard of care (SOC), i.e., a “status quo” situation.

Studies suggest that many potential contraception users lack access due to poverty [7, 34]. For example, the price of a copper implant in Uganda ranges from $ 6 -$12 [35]. Most of the service providers in Kampala’s urban slums are private health facilities that charge up to four times higher than public facilities [36]. This is in the context of an urban population where most inhabitants live on less than a dollar per day [37]. The study will add to the evidence base to support the economic efficiency of universal access to contraception in low-income countries in general and marginalized populations in particular.

## Methods

### Design

We conducted a model-based cost-effectiveness analysis using data from a one-year prospective impact evaluation of the FPBC intervention conducted in Uganda. Data on costs and outcomes were obtained from this evaluation, which was a quasi-experimental study conducted in the urban slums of Kampala [38]. Additional data were obtained from the published literature.

### The family planning benefits card program

The FPBC program was a one-year pilot project, a novel incentive system intended to increase uptake of contraception services among adults aged 18 to 30 in the Kamwokya slum area of Kampala, Uganda’s capital city [4, 38]. The program provided a card that allowed access to free male and female condoms, implants, intrauterine devices (IUDs), injections, vaginal rings, contraceptives and emergency contraceptive pills, diaphragms, counselling and guidance, and pregnancy and HIV testing. The incentives were given to partner clinics to boost contraception provision in the form of subsidized prices to all administered contraception methods compared to the normal market prices. The FPBC was found to be highly acceptable and was utilized to a significant extent in this population [28].

### Target population

The reference case population in the current study included young female adults aged 18 to 30 years, non-users of modern contraception methods, sexually active and not pregnant, and willing to give informed consent [4, 38]. This criterion was the basis for eligibility for the prospective study as well as the current study. Therefore, this study reported outcomes and costs on a per-person basis.

### Setting

The study setting and context were urban slums located in the city suburbs of Kampala, Uganda’s capital city. These two slums were purposively selected to assess the effectiveness of the FPBC due to their similar socio-demographic characteristics—the largest population is unemployed, falls in the lowest wealth quintile, and has the highest unmet need for contraception (30%) [3, 37, 39]. The current study findings may be applied in informing health policy in similar urban slum settings in low-income countries.

### Comparators

We compared the FPBC with the SOC, considered as a status quo situation, an out-of-pocket venture, where the young female adults visited clinics or health facilities of their choices and paid for all the services requested and provided. Baseline and endline data for the SOC was collected at the same time as in the intervention group, and the intention-to-treat analysis was applied to estimate the intervention effect.

### Time horizon

The time horizon of the analysis was one-year, consistent with the length of the prospective study from which cost data came [38].

### Decision analytic model

The decision-analytic model (decision tree) was developed to follow a young female adult’s likely course of action following the FPBC intervention. Data to parameterize the model were obtained from the one-year pilot extensive study [40] and published literature [30, 32]. The decision node represents the choice between the FPBC program and the SOC. [38] After being selected to the FPBC intervention, young women were divided into those who received the FPBC and those who did not. Young women who received the card were further divided into those who used it to access modern contraception methods and those who did not. Young women who accessed modern contraception methods using the card were also divided into those who suffered the adverse effects (the negative side effects of using modern contraception methods, such as mood change, low sex drive, weight gain, amenorrhea, bleeding, etc) and those who did not. It should be noted that some young women in the FPBC did not receive cards due to logistic reasons and delayed consenting. Thus, we made a conservative assumption that those who missed cards accessed modern contraception through out of pocket similar to the SOC arm, but with a triage path of an FPBC beneficiary. Further still, some of the card beneficiaries decided not to use the cards due to the fear of the side effects of modern contraception methods and the desire to conceive.

In the control arm (SOC), young women were divided into those who paid out of pocket to access modern contraception methods and those who did not. These were further divided into those who suffered adverse effects due to modern contraception methods and those who did not.

**Fig. 1:**
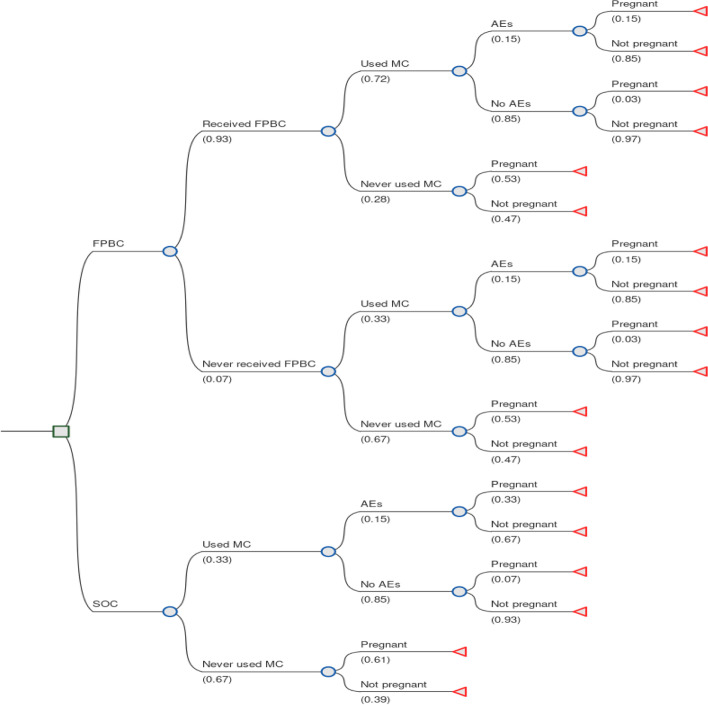
Decision tree showing the impact of the FPBC. A circle shows a chance node (the probability of an event occurring), a square shows the decision node (the probability of either going for the new intervention [FPBC] or the SOC), and a triangle shows an end node

In both the FPBC and SOC, the end node represents the proportion of young women who reported unintended pregnancies at the end of the study [40]. Figure 1 below shows a schematic structure of the decision tree model.

### Outcomes

This study measured the probability of unintended conception (pregnancy) as the main outcome.

#### Pregnancy

This study did not treat normal pregnancies as a burden or a bad thing but rather looked at unintended pregnancies as a burden to the families and societies due to their negative impact on economic growth, economic development, and public health [41, 42]. The study used the proportions of young female adults who reported having unintended pregnancies – mistimed or unwanted with no desire of having children— at the end of the study to estimate the probability of conception in the two groups. From the intention-to-treat analysis, young women who reported being pregnant at the end of six months (the follow-up period) were further stratified into: 1) pregnant without using modern contraception, 2) pregnant while using modern contraception and experiencing adverse effects, and 3) pregnant while using modern contraception without experiencing any adverse effects.

### Perspective

The analysis was conducted from both the modified societal and provider (government of Uganda through the Ministry of Health and other non-government organizations) perspectives. The ministry of health covers only 10% of the contraception budget [43]; 90% is catered for by non-government organizations and individuals through out-of-pocket expenditure. Therefore, considering the provider and modified societal perspectives would include all direct medical costs and indirect costs incurred by individuals, as well as direct non-medical costs incurred by providers.

### Costs

Direct medical costs (personnel time, training costs, laboratory tests, drugs, medical devices, and supplies), direct non-medical costs (administration, overhead, utilities) were analyzed to suit the provider (in this case, Ministry of Health) perspective. In contrast, costs related to lost productivity were added to cater for the modified societal perspective. We conducted key informant interviews (KIIs) with the medical personnel from partner clinics and the FPBC beneficiaries to estimate the time spent administering each modern contraception method, and the waiting time at the clinics, respectively. These KIIs were conducted on an appointment basis, where the medical personnel and FPBC beneficiaries were notified about the purpose and location of the meeting with the help of community health workers (CHWs), and each session lasted for 15 to 20 minutes. The average time for administering each modern contraception method was estimated from the recorded notes, as well as the average waiting time by the FPBC beneficiaries. These time estimates were multiplied by the average wage in both public and private sectors [44].

The beneficiaries’ occupations and the average waiting time were key components in estimating the lost productivity costs (indirect costs) [40]. Costs on the medical supplies and drugs were estimated from the Management Sciences for Health (MSH) price catalogues [45] since the prices paid to the partner clinics by the insurance company embedded an incentive component, thus a slightly higher price. Costs on equipment, including cars and computers, were annuitized at an annual discount rate of 3% to account for depreciation. Other direct medical (salaries for personnel, training, and medical supplies) and non-medical (administration, program costs, and overhead) costs were estimated by reviewing account records from the GHE database using the line-by-line cost estimation method [46].

The primary study did not incur any other cost in the SOC group except for the research related costs, which were not considered, but these costs were estimated based on the reported proportions of usage of different modern contraception methods at the end of the study [40]. Like in the FPBC, these proportions were also used to estimate medical personnel time, childcare costs, drugs, and medicine. Indirect costs were estimated based on the participants’ reported occupations multiplied by the average waiting time. Other costs, such as personnel and overhead costs, were assumed to be similar, and the SOC did not incur any program-related costs (administration), as shown in Table 1.

**Table 1:** Itemized costs (2022 $US) and proportions used in the analysis

Cost category^**a**^	Per person cost [Low, High]		Reference
	FPBC	SOC	
Personnel (FPBC)	116 (58-174)	116 (58-174)	Primary study
Medical personnel time	2 (1-3)	2 (1-3)	Primary study
Training CHWs	9 (4-13)	4 (2-6)	Primary study
Administration	25 (13-38)	–	Primary study
Equipment	4 (2-7)	4 (2-6)	Primary study
Overhead	37 (19-56)	37 (19-56)	Primary study
Childcare	–	–	Primary study
Lost productivity	2 (1 - 3)	1 (0.5 - 1.5)	Primary study
**Proportion of users for each modern contraception method, %**^**b**^			
Emergency Contraception	0.06 (0.04-0.10)	0.01 (0.02 - 0.33)	Primary study
Implants	0.17 (0.13-0.22)	0.07 (0.04 - 0.12)	Primary study
Injectables	0.43 (0.37-0.49)	0.52 (0.46 - 0.58)	Primary study
IUDs	0.08 (0.05-0.12)	0.01 (0.02 - 0.33)	Primary study
Male condoms	0.04 (0.02-0.07)	0.08 (0.05 - 0.12)	Primary study
Pills	0.21 (0.16-0.27)	0.30 (0.25 - 0.36)	Primary study

^a^Cost ranges represent +/− 50% of each cost

^b^Proportion ranges are based on the lower and upper boundary of the 95% confidence interval

Overall, in both the FPBC and SOC, the summation of these costs was multiplied by the proportion of respondents who used different modern contraception methods to determine the cost related to each contraception method.

### Currency, price date, and conversion

All costs were adjusted for inflation to cater for the exchange rate fluctuations using the bank of Uganda exchange rates [47] and consumer price indices for Uganda [48] since these data were obtained from several years, i.e., 2017, for both the FPBC and SOC. All costs were reported in 2022 US dollars.

### Analysis

In the base-case analysis, we compared the average cost per young female adult from the modified societal and payer perspectives, the probability of conception, and the ICER using the cost per unintended pregnancy averted. This enabled us to identify the dominant and dominated interventions between the FPBC and the SOC.

We conducted the deterministic (one way) sensitivity analysis to identify model parameters that most influenced the ICERs. Since there was no data on 95% confidence intervals on cost parameters, a range of +/− 50% was applied [30, 32]. Monte Carlo simulation was employed to generate 1000 iterations to calculate the expected outcome values and perform the probabilistic sensitivity analysis to assess further the impact of uncertainties surrounding key model parameters on the ICERs and the results’ robustness. A beta distribution was assumed for all probabilities while a normal distribution was applied to all costs, assuming that they were normally distributed [49, 50]. All analyses were conducted in Microsoft Excel, and as much as possible, this study followed the Consolidated Health Economic Evaluation Reporting Standards (CHEERS) statement [51]. Table 2 shows the different model parameters with their probability distributions.

**Table 2:** Parameters in the Decision Tree Model

Input parameters	Value	Probability distribution	Reference
**Probabilities, %**			
Entered the FPBC program	0.93	β(∞ = 196.76; β = 14.81)	Primary study
Used the FPBC to access Modern contraception	0.72	β(∞ = 136.7; β = 53.16)	Primary study
Adverse effects of modern contraception^a^	0.15	β(∞ = 21.10; β = 119.57)	Primary study
Pregnant while using modern contraception with AEs (FPBC)	0.15	β(∞ = 21.10; β = 119.57)	Primary study
Pregnant while using modern contraception without AEs (FPBC)	0.03	β(∞ = 5.56; β = 179.68)	[52]
Pregnant while not using modern contraception (FPBC)	0.53	β(∞ = 124.46; β = 110.37)	Primary study
Used modern contraception (SOC)	0.33	β(∞ = 80.74; β = 163.93)	Primary study
Pregnant while using modern contraception with AEs (SOC)	0.33	β(∞ = 80.74; β = 163.93)	Primary study
Pregnant while using modern contraception without AEs (SOC)	0.07	β(∞ = 14.81; β = 196.76)	[52]
Pregnant while not using modern contraception (SOC)	0.61	β(∞ = 160.63; β = 102.70)	Primary study
**Costs, $**^**b**^			
Direct Medical Costs (FPBC)	127	Normal (64;191)	Primary study
Direct Non-Medical Costs (FPBC)	66	Normal (33;99)	Primary study
Indirect costs (FPBC)	2	Normal (1;3)	Primary study
Direct Medical Costs (SOC)	122	Normal (61;183)	Primary study
Direct Non-Medical Costs (SOC)	41	Normal (21;62)	Primary study
Indirect costs (SOC)	1.00	Normal (0.5;1.5)	Primary study

^a^Sensitivity ranges represent +/−50% for cost parameters

^b^Assumed to be identical in both the FPBC and SOC

*AEs* adverse effects, *FPBC* family planning benefits card, *SOC* standard of care

### Approach to engagement with beneficiaries and others affected by the study

Besides engaging with the medical personnel from partner clinics and the FPBC beneficiaries to estimate the time spent administering each modern contraception method and the waiting time at the clinics, their contribution to the modelling process was considered, particularly in generating decision tree branches.

### Ethical considerations

The respective institutional review boards approved the current study—Mbarara University of Science and Technology [MUREC1/7 No. 10/05-17], the Uganda National Council for Science and Technology, and other local authorities.

All study participants were initially sensitized about the purpose of the study with the help of CHWs and provided informed consent before being recruited to participate in the study.

## Results

### Base case analysis

Table 3 shows the results summary from the base case analysis. The average probability of unintended conception was lower in the FPBC than in the SOC (0.20 vs. 0.44).

**Table 3:** Base case results showing the average cost (per young woman), incremental costs, probability of conception and ICERS comparing the FPBC to the SOC

	FPBC		SOC	
	Societal	Provider	Societal	Provider
Absolute costs ($)	195	193	164	163
Probability of conception	0.2	–	0.44	–
Incremental cost	31	30	–	–
ICER per unintended pregnancy averted	125	121	–	–

*ICER* Incremental Cost-effectiveness Ratio, *FPBC* Family Planning Benefits Card, *SOC* Standard of Care

The average societal cost per young woman was higher for the FPBC from the modified societal perspective ($195 vs. $164) and the provider perspective ($193 vs. $163). The absolute ICER comparing the FPBC to the SOC was $125 per unintended pregnancy averted from the societal perspective and $121 from the provider perspective.

### Sensitivity analyses

**Fig. 2:**
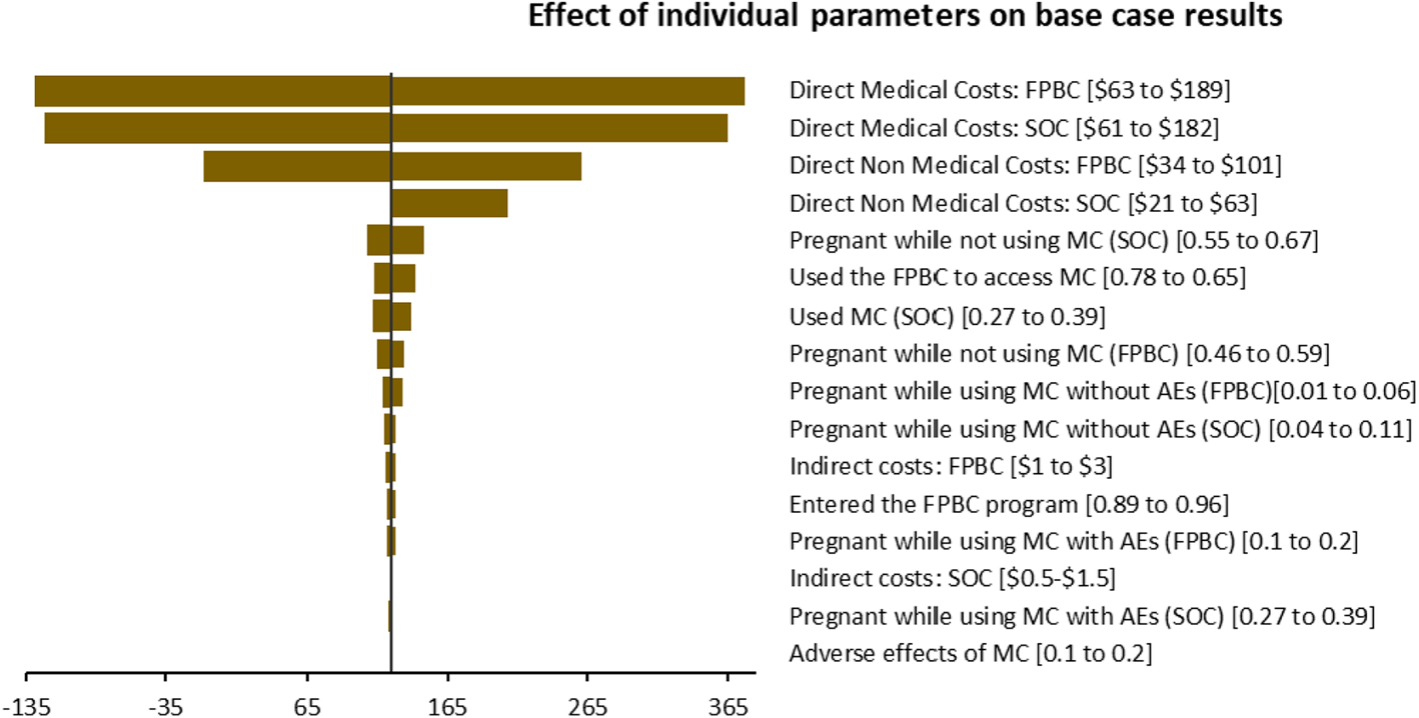
Tornado diagram of one-way sensitivity analysis from the modified societal perspective. Different variables and their uncertainty impact on the ICERs per unintended pregnancy averted are shown. AEs, adverse effects; MC, modern contraception

As shown in Fig. 2, the deterministic sensitivity analysis indicated that the ICERs were most sensitive to the uncertainties surrounding the direct medical costs and direct non-medical costs related to the FPBC and SOC, and the probability of pregnancy while not using modern contraception and using the FPBC to access modern contraception. The ICERs remained within plausible ranges of the cost-effectiveness threshold, thus robust to sensitivity analyses (Fig. 2).

**Fig. 3:**
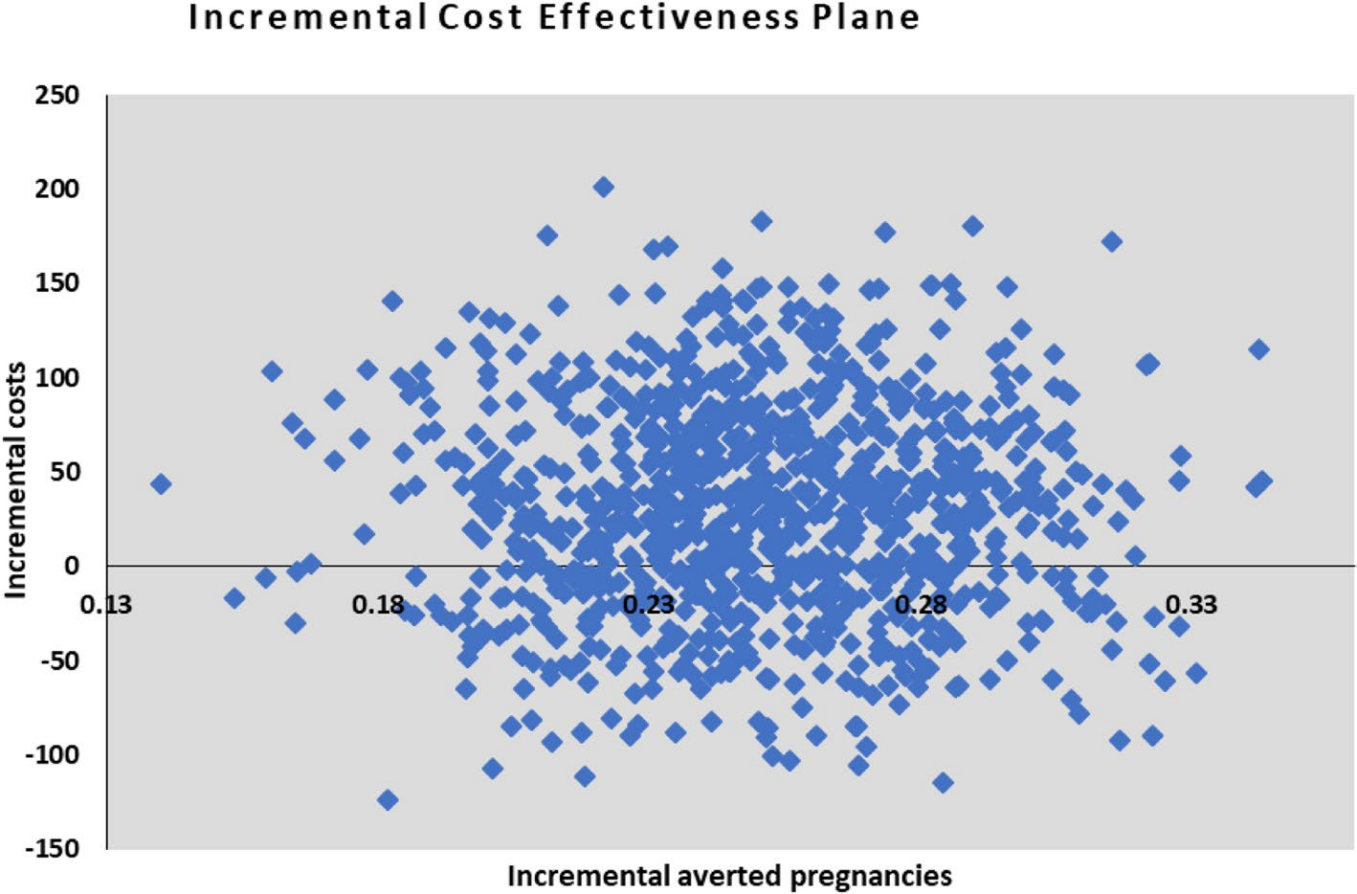
Incremental Cost-effectiveness scatter plot obtained from the probabilistic sensitivity analysis showing the distribution of pairs for incremental costs and averted pregnancies on the cost-effectiveness plane. The largest distribution is in the northeast quadrant and fairly distributed in the southeast quadrant

In Fig. 3, the incremental cost-effectiveness plane from the probabilistic sensitivity analysis through Monte Carlo simulation showed that most cost-effectiveness pairs were distributed in the northeast and southeast quadrants of the cost-effectiveness plane. The widespread pair points across the cost-effectiveness plane indicate a high level of certainty that the FPBC is more cost-effective than the SOC, but with some uncertainty on whether the FPBC is less costly than the SOC.

**Fig. 4:**
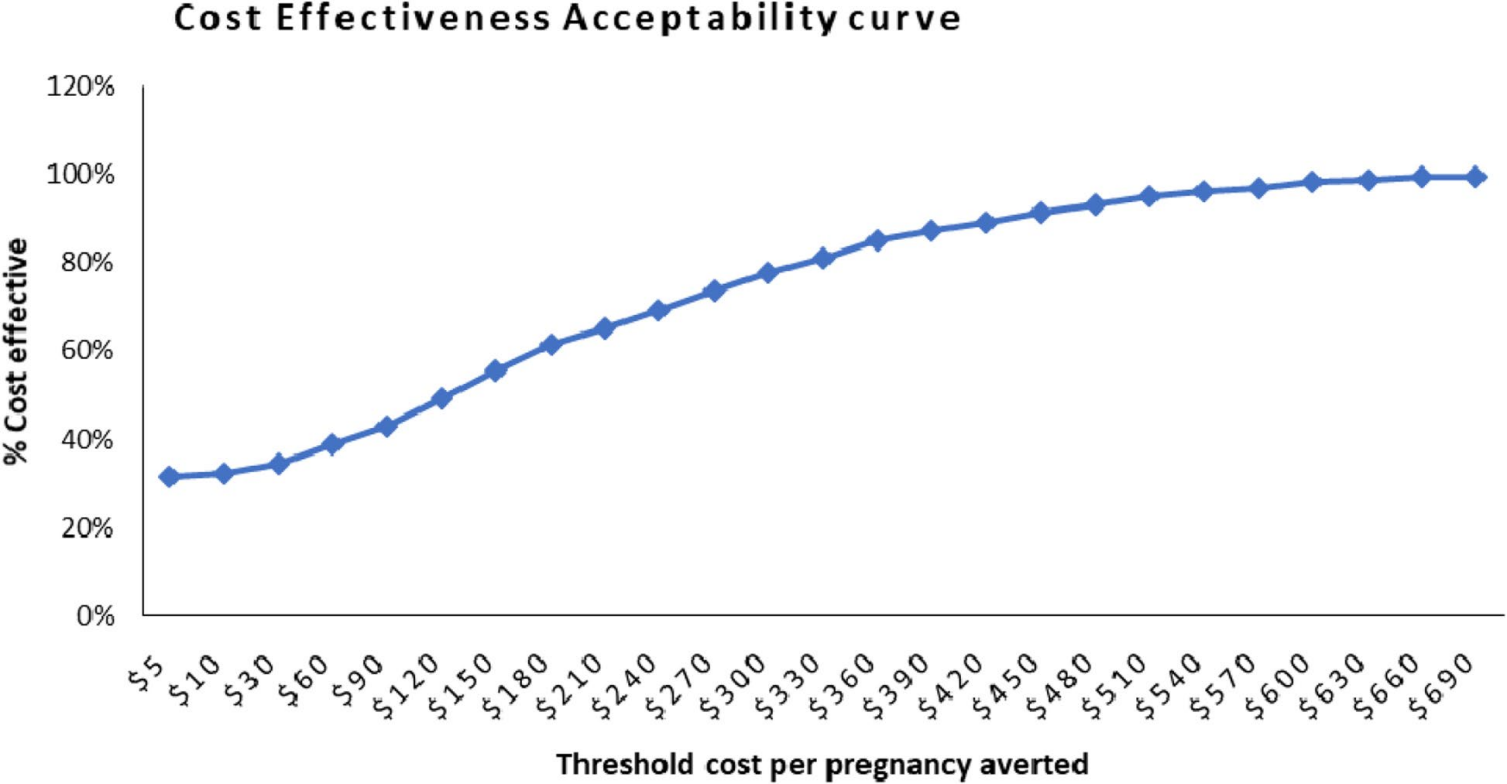
Cost-effectiveness acceptability curves extracted from the probabilistic sensitivity analysis. The curve indicates the probability of the FPBC being cost-effective compared to the SOC for a sample of 1000 simulations over a range of willingness to pay (threshold) values

In Fig. 4, the cost-effectiveness acceptability curve indicates the probability that the FPBC is more cost-effective than the SOC at a certain range of willingness to pay values curbed at 3 times Uganda’s GDP per capita, the gold standard for LMICs. It suggests that the percentage of iterations in which the FPBC is more cost-effective than the SOC nears 100% at a cost-effectiveness threshold of less than $1046, the GDP per capita of Uganda.

## Discussion

This study used a decision tree model and data from a quasi-experimental study design to assess the one-year cost-effectiveness of the FPBC compared to the SOC. The results showed that the FPBC was highly cost-effective –largely dominated the SOC—from both the modified societal perspective and the provider perspective. In other words, the FPBC led to increased costs, and a lower probability of unintended conception for one year compared to the SOC. The difference in the annual probability of conception between the two groups (FPBC Vs. SOC) was considerable at 24%. Arguably, this can be attributed to contraception failure since most young women in the SOC used short-term contraception methods with a reported failure rate of about 3% [52]. In addition, the low probability of conception in the FPBC could be explained by the large proportions of users who entered the FPBC program (93%) and used the FPBC to access modern contraception (72%), particularly long-term methods, such as implants, and IUDs. Notably, these results were robust to both the deterministic and the probabilistic sensitivity analyses, i.e., there were no significant changes in the results—high costs and better outcomes—in favour of the FPBC like in the base case analysis.

In the deterministic sensitivity analysis, the ICERs were most driven by the uncertainties surrounding the direct medical costs, and direct non-medical costs related to the FPBC and SOC, as well as the probability of pregnancy while not using modern contraception and using the FPBC to access modern contraception. The most significant component of direct medical costs were the personnel costs (60%), while program costs largely contributed to direct non-medical costs (13%). The large program costs could be attributed to the fact that the FPBC model gave incentives to partner clinics in the form of higher prices than the normal prices, as a motivation to provide free services to all FPBC beneficiaries. Perhaps if these incentives were not huge or if it were an entirely free service venture, the FPBC would even be cheaper. Additionally, the high indirect costs related to the FPBC can be explained by the fact that many FPBC beneficiaries opted for long term contraception methods like IUDs, and implants, which required a lot of administration time by the service providers—the time spent on administering different methods by the healthcare service providers was a key component in estimating indirect costs. A cost consequences analysis study on induced abortion in Uganda reported similar results, i.e., the largest component of both the societal and provider costs were direct medical and indirect costs [53].

Our results are consistent with the considerable literature on non-incentive-based contraception interventions in other LMICs, including Nigeria, Mexico, India, Afghanistan, Vanuatu and the Solomon Islands [24–28], which were found to be cost-effective. Similarly, these results agree with results from several non-incentive-based contraception interventions in Uganda that proved highly cost-effective and cost-effective [29–33]. Such interventions included; the universal access of contraception among all women in Uganda using hypothetical interventions [$ 105 per DALY averted and $ 629 per DALY averted, respectively] [30, 32], actual contraception methods such as injectables [$ 2.94 per birth averted], condoms [$ 2.06 per birth averted] and oral contraceptives [$ 1.65 per birth averted] [31], and a community-based provision vs. facility-based provision in a non-government organization setting [$ 21.21 per couple year of protection vs. $37.7 per couple year of protection, respectively] [33].

In dominating the SOC, the FPBC matches up with other incentive-based healthcare approaches and interventions that are cost-effective in Uganda, such as vouchers for treatment of sexually transmitted infections and maternal health [$ 302 per DALY averted] [54], vouchers for maternal health services with transport facilitation at any health facility in a rural setting [$ 302 per DALY averted] [55] and a savings scheme among adolescents orphaned by AIDS [$ 267 per 0.2 standard deviation range] [56]. It should be noted that the FPBC program structure was conceptually similar to an insurance model that thrives on significant economies of scale with higher numbers. And, given the successful proof of concept, feasibility (high acceptability and utilization) [4], and effectiveness [40], the marginal cost of the FPBC deployment (cost per additional user) would substantially reduce. This implies that the program and administration costs are incurred upfront and considered “sunk” costs. With this consideration, the ICERs could drop substantially and would be considered cheaper in a low-income setting. This may potentially be an area for future research to ascertain these results using different approaches.

Besides, the implementation of these interventions, including the FPBC, entirely depends on policymakers. The FPBC could potentially lead to increased savings, reduced mortalities, and contribute towards long-life span, attaining the SDGs and economic growth and development in the long run. The FPBC could also be used as one of the panaceas to the worldwide gross inequalities in accessing modern contraception since it covers the financial risk of out-of-pocket expenditure on modern contraception among marginalized groups [8–14].

### Study limitations

Like other models, the current analysis could not elude from limitations, which emanate from data availability issues and assumptions. By conducting interviews with the FPBC beneficiaries to estimate indirect costs (lost productivity) after one year of the program closure, we likely overestimated the costs due to recall bias. We also could have underestimated the actual costs by assuming that the personnel and overhead costs in the SOC group were similar to the FPBC. Nevertheless, we believe that the effect was not too huge to affect the ICER estimates.

We conducted the analysis from both the modified societal and provider perspectives, and the outcomes data were adopted from a quasi-experimental study—conceptually similar to a randomized controlled trial using the intention-to-analysis technique. Therefore, our results are generalizable and applicable to other low-and middle-income settings.

## Conclusion

The FPBC is a highly cost-effective intervention in the short-term compared to the SOC, and with greater certainty that it can be favourably cheaper in the long run due to the low marginal costs of deploying additional FPBCs. This bodes well towards the implementation of the FPBC to increase access to modern contraception methods among young women living in slums and other vulnerable groups, as Uganda transitions towards achieving five of the seventeen 2030 SDGs through the reduction of unintended pregnancies, infant and maternal mortalities, as well as having well-planned for children.

## Data Availability

All the data used and presented in this study are available upon request.

## Abbreviations

CHEERS: Consolidated Health Economic Evaluation Reporting Standards
CHWs: Community health workers
DALYs: Disability-adjusted life years
FPBC: Family planning benefits card
GDP: Gross domestic product
ICER: Incremental cost-effectiveness ratio
IUDs: Intrauterine devices
KIIs: Key Informant interviews
LMICs: Low-and middle-income countries
MSH: Management Sciences for Health
SGDs: Sustainable development goals
SOC: Standard of care
WHO: World Health Organization
